# Erectile dysfunction is frequent in systemic sclerosis and associated with severe disease: a study of the EULAR Scleroderma Trial and Research group

**DOI:** 10.1186/ar3748

**Published:** 2012-02-20

**Authors:** Chingching Foocharoen, Alan Tyndall, Eric Hachulla, Edoardo Rosato, Yannick Allanore, Dominique Farge-Bancel, Paola Caramaschi, Paolo Airó, Starovojtova M Nikolaevna, José António Pereira da Silva, Bojana Stamenkovic, Gabriela Riemekasten, Simona Rednic, Jean Sibilia, Piotr Wiland, Ingo Tarner, Vanessa Smith, Anna T Onken, Walid Ahmed Abdel Atty Mohamed, Oliver Distler, Jadranka Morović-Vergles, Andrea Himsel, Paloma Garcia de la Peña Lefebvre, Thomas Hügle, Ulrich A Walker

**Affiliations:** 1 Department of Rheumatology, Basel University, Burgfelderstrasse 101, Basel 4012, Switzerland; 2 Department of Internal Medicine, Hôpital Claude Huriez, Place de Verdun, Lille 59035, France; 3 Centro per la Sclerosi Sistemica - Dipartimento di Medicina, Università 'La Sapienza', Viale del Policlinico 155, Rome 00185, Italy; 4 Rhumatologie A, Hôpital Cochin, Université Paris Descartes, Saint-Vincent-De-Paul La Roche-Guyon 27, rue du Fg Saint-Jacques, Paris 75679, France; 5 Department of Internal Medicine, Hospital Saint Louis, 1 avenue Claude Vellefaux, Paris 75010, France; 6 Rheumatology Unit, University of Verona, Piazzale LA Scuro 10, Verona 37134, Italy; 7 Rheumatology and Clinical Immunology Service, Spedali Civili di Brescia, P. le Spedali Civili 1, Brescia 25123, Italy; 8 Institute of Rheumatology, Russian Academy of Medical Science, Kashirskoye Shosse, 34 A, Moscow 115522, Russia; 9 Hospitais da Universidade, Coimbra 3000-075, Portugal; 10 Institute for Prevention, Treatment and Rehabilitation of Rheumatic Disease, Srpskih Junaka 2, Niska Banja 18205, Serbia and Montenegro; 11 Department of Rheumatology, Charité University Hospital, Schumannstraße 20/21, Berlin 10117, Germany; 12 Rheumatology Clinic, University of Medicine & Pharmacy 'Luliu Hatieganu' Cluj, Str. Clinicilor nr. 2-4, Cluj-Napoca 400006, Romania; 13 Department of Rheumatology, University Hospital of Strasbourg, 1 avenue Molière 83049, Strasbourg 67098, France; 14 Department of Rheumatology and Internal Diseases, Wroclaw University of Medicine, Ul. Borowska 213, Wroclaw 50-556, Poland; 15 Department of Rheumatology and Clinical Immunology, Justus-Liebig-University, Benekestraße 2-8, Bad Nauheim 61231, Germany; 16 Department of Rheumatology, University of Ghent, De Pintelaan 185, Ghent 9000, Belgium; 17 Department of Dermatology and Allergy, TU Munich, Biedersteiner Straße 29, Munich 80802, Germany; 18 Unit of Rheumatology, Alexandria University Student Hospital, Champlion Square Mazarita, Alexandria, Egypt; 19 Department of Rheumatology, University Hospital Zurich, Gloriastrasse 25, Zurich 8032, Switzerland; 20 Division of Clinical Immunology and Rheumatology, University Hospital Dubrava, Av. G. Šuška 5, Zagreb 10000, Croatia; 21 Department of Rheumatology, Johann Wolfgang Goethe University, Theodor-Stern-Kai 7, Frankfurt am Main 60590, Germany; 22 Hospital Madrid Norte, c/OÑA N° 10, Madrid 28050, Spain

## Abstract

**Introduction:**

Erectile dysfunction (ED) is common in men with systemic sclerosis (SSc) but the demographics, risk factors and treatment coverage for ED are not well known.

**Method:**

This study was carried out prospectively in the multinational EULAR Scleroderma Trial and Research database by amending the electronic data-entry system with the International Index of Erectile Function-5 and items related to ED risk factors and treatment. Centres participating in this EULAR Scleroderma Trial and Research substudy were asked to recruit patients consecutively.

**Results:**

Of the 130 men studied, only 23 (17.7%) had a normal International Index of Erectile Function-5 score. Thirty-eight per cent of all participants had severe ED (International Index of Erectile Function-5 score ≤ 7). Men with ED were significantly older than subjects without ED (54.8 years vs. 43.3 years, *P *< 0.001) and more frequently had simultaneous non-SSc-related risk factors such as alcohol consumption. In 82% of SSc patients, the onset of ED was after the manifestation of the first non-Raynaud's symptom (median delay 4.1 years). ED was associated with severe cutaneous, muscular or renal involvement of SSc, elevated pulmonary pressures and restrictive lung disease. ED was treated in only 27.8% of men. The most common treatment was sildenafil, whose efficacy is not established in ED of SSc patients.

**Conclusions:**

Severe ED is a common and early problem in men with SSc. Physicians should address modifiable risk factors actively. More research into the pathophysiology, longitudinal development, treatment and psychosocial impact of ED is needed.

## Introduction

Systemic sclerosis (SSc) is a connective tissue disorder in which vascular alterations and endothelial damage are prominent and lead to progressive and widespread dysfunction of various organs. Vascular symptoms such as Raynaud's phenomenon, digital ulcers and pulmonary arterial hypertension are also a frequent target of diagnostic and therapeutic efforts [1]. Men with SSc may develop erectile dysfunction (ED), a vascular complication that is not frequently addressed in studies. Owing to the predominance of the female gender in SSc, studies of ED in SSc men are more difficult to perform. The available data from small studies have suggested that ED is more common in SSc than in the normal population and in other autoimmune diseases [2-4]. ED has been attributed to a vascular process with diminished arterial blood supply of the corpus cavernosum [5-8], corporeal fibrosis and accumulation of extracellular matrix [5-7].

This study aims to confirm the high prevalence using an unprecedentedly large multicentre cohort, and to describe hitherto unaddressed SSc characteristics (autoantibody status, SSc subtype, disease duration) of men with ED, to study SSc-related complications and nonrelated comorbidities as factors in the development of ED, and to report on current treatment regimens.

## Materials and methods

### Data collection

The study was performed using the multinational database of the EULAR Scleroderma Trial and Research (EUSTAR) group, which was inaugurated in 2004. Participating centres are required to have local ethics committee approval; patients must provide informed written consent prior to entry into the Minimal Essential Data Set [9]. Patients must fulfil the American College of Rheumatology classification criteria for SSc. For the purpose of this study, the Minimal Essential Data Set online electronic data-entry system - which prospectively follows patients on yearly visits - was amended by a separate data-entry page with items specific to the ED study. EUSTAR centres intending to participate in the ED study were displayed in this separate data-entry system and were asked to provide all men consecutively with the International Index of Erectile Function-5 (IIEF-5), a self-administered questionnaire that is validated in several languages, has high retest reliability, and has demonstrated sensitivity and specificity for detecting treatment-related changes [10]. The IIEF-5 provides a numerical score that is classified into five categories: severe ED (scores 5 to 7), moderate ED (scores 8 to 11), mild to moderate ED (scores 12 to 16), mild ED (sores 17 to 21), and no ED (scores 22 to 25).

In addition to the IIEF-5 instrument, men were asked to provide information about the time of ED onset and the use of phosphodiesterase-5 inhibitors for the specific purpose of ED treatment (not for pulmonary hypertension), as well as intraurethral or intracavernous prostaglandin preparations. Physicians were also questioned about factors known to increase the risk for ED, such as hypercholesterolaemia, diabetes mellitus, stroke, smoking, peripheral macroangiopathy and coronary heart disease.

### Statistical analysis

The dataset was analysed using Stata version 11.0 (StataCorp Inc., College Station, TX, USA). SSc presentations were analysed cross-sectionally for associations between ED and other clinical features of ED. Continuous data were presented as the mean (± standard deviation) or the median with interquartile range (IQR) as appropriate, while binary parameters were presented as percentages. Odds ratios with 95% confidence intervals and linear regressions were calculated to estimate effect sizes. Variables with *P *< 0.1 were then entered into a multivariate logistic regression model.

## Results

### Participants

Twenty-two EUSTAR centres in 13 countries participated in this study, which started in October 2009. These EUSTAR centres were prospectively following 2,469 women and 463 men (gender ratio 5.3:1). At the time of census in May 2011, the centres had recruited a total of 130 men for this study. A comparison between study participants and nonparticipants demonstrates that the participants were representative of the male population in the EUSTAR centres with respect to important demographic parameters and disease characteristics such as age, antinuclear antibodies, and disease duration (Table 1). Participants had a higher proportion of puffy hands and digital ulcers, as well as a higher modified Rodnan skin score, but less frequently an impairment of the diffusion capacity of the lung for carbon monoxide below 80% of normal. Differences in systolic pulmonary arterial pressure estimated by echocardiography were statistically significant but medically less relevant.

**Table 1:** Characteristics of men that were included in the ED substudy by the 22 participating centres compared to those not included.

	Nonparticipants (*n *= 463)	Participants (*n *= 130)	*P *value
Age (years)	54.3 (46.0 to 64.1)	52.3 (45.1 to 61.5)	0.12
SSc duration by Raynaud's phenomenon (years)	6.4 (3.4 to 11.7)	7.0 (3.7 to 11.9)	0.21
SSc duration by first non-Raynaud's symptom (years)	5.4 (2.7 to 9.2)	6.0 (3.0 to 10.3)	0.20
Topoisomerase I (Scl70)-autoantibodiespositive	42.8	46.7	0.44
Anti-centromere autoantibodies-positive	14.9	15.7	0.82
U1RNP-positive	6.5	4.7	0.51
RNA polymerase III-positive	9.4	2.8	0.08
Diffuse SSc	56.8	63.7	0.18
Puffy hands	34.3	54.6	< 0.001*
mRSS	10 (4 to 16)	13 (6 to 20)	0.01*
mRSS > 20	16.8	25.0	0.05
C-reactive protein elevation	33.6	24.5	0.07
Digital ulcers	34.7	44.0	0.04*
Synovitis	13.1	20.0	0.05
Joint contractures	33.0	31.8	0.81
Tendon friction rubs	10.2	10.0	0.94
Muscle weakness	22.2	20.9	0.76
Muscle atrophy	11.1	15.5	0.17
Creatine kinase elevation	11.6	13.3	0.58
Oesophageal symptoms	54.4	60.4	0.23
Stomach symptoms	16.0	13.5	0.50
Intestinal symptoms	18.8	12.6	0.11
Lung fibrosis on chest X-ray or HRCT	60.4	56.7	0.59
Forced vital capacity (% predicted)	89 (75 to 101)	92 (81 to 103)	0.36
Forced vital capacity < 80%	32.2	23.3	0.10
DLCO (% predicted)	66 (49 to 82)	69 (55 to 88)	0.05
DLCO < 80%	71.	59.1	0.01*
PAPsys (mmHg)	30 (25 to 37)	27 (20 to 36)	0.004*
PAPsys > 40 mmHg	22.3	19.4	0.51
Diastolic dysfunction	20.4	18.8	0.70
Pericardial effusion	7.1	5.7	0.64
Left ventricular ejection fraction < 60%	25.1	20.0	0.31
Arterial hypertension	21.5	23.6	0.75
Renal crisis	3.3	4.5	0.50
Proteinuria	8.6	9.4	0.79
EULAR SSc activity score	1.5 (0.5 to 3.0)	2 (0.5 to 3.5)	0.27
High SSc activity (score ≥ 3)	27.2	35.2	0.19
Hypocomplementaemia	5.0	5.9	0.71

Demographic parameters and disease characteristics from the EULAR Scleroderma Trial and Research database

Data presented as median (interquartile range) or percentage. DLCO; diffusion capacity of the lung for carbon monoxide; HRCT, high resolution computed tomography; mRSS; modified Rodnan skin score; PAPsys; systolic pulmonary arterial pressure; SSc, systemic sclerosis. *Significant at *P *< 0.05.

### Prevalence of erectile dysfunction in systemic sclerosis

**Figure 1:**
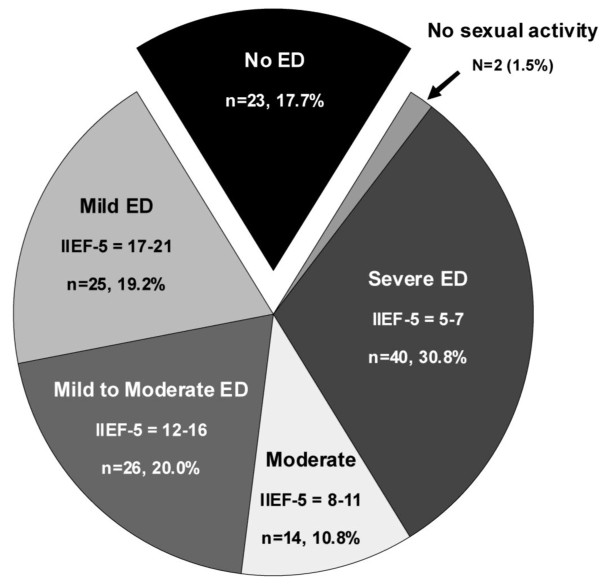
**Prevalence and severity of erectile dysfunction among the 130 participants**. ED, erectile dysfunction; IIEF-5, International Index of Erectile Function-5.

Of the 130 participants, only 23 men (17.7%) had a normal IIEF-5 score (≥ 22). Two men had not engaged in any sexual activity in the 6 months prior to filling out the IIEF-5 questionnaire and could therefore neither be attributed to the ED group or to the non-ED group. The remaining 105 men (81%) had variable degrees of ED. The largest group of all participants (38%) had severe ED (Figure 1). The median IIEF-5 score of all SSc patients was 13 (IQR 6 to 19). Among the men with ED, the median IIEF-5 score was 11 (IQR 5 to 16).

### Comorbidities

A number of conditions are associated with ED in the general population. These conditions include cardiovascular risk factors, medications (antidepressants, sedatives, neuroleptics, antiepileptics, diuretics), alcoholism, neurological and endocrine disorders, as well as prostatic disease [8,11,12]. The majority of the participating men had at least one such comorbidity (Table 2). Men with ED more frequently had more than one simultaneous comorbidity than men with normal erections.

**Table 2:** Comorbidities of the participants

	No erectile dysfunction (*n *= 23)	Erectile dysfunction (*n *= 105)	Odds ratio (95% confidence interval)
Cardiovascular risk factors			
Systemic arterial hypertension	14.3	24.1	1.11 (0.92 to 1.34)
Diabetes mellitus	4.4	6.9	1.08 (0.82 to 1.42)
Coronary heart disease	4.4	13.3	1.17 (0.98 to 1.39)
Hypercholesterolaemia	19.1	13.3	0.92 (0.70 to 1.21)
History of smoking	31.8	42.6	1.09 (0.92 to 1.29)
Cigarette smoking (pack-years)	15 (10 to 21)	20 (9 to 30)	*P *= 0.69
Medication			
Antidepressant, sedative, neuroleptic or antiepileptic	4.4	9.1	1.12 (0.89 to 1.40)
Thiazides or spironolactone	4.4	7.0	1.08 (0.82 to 1.43)
Alcohol consumption (> 2 units/day)	0	13.7	1.27 (1.15 to 1.40)*
Other			
Depression	4.6	9.0	1.11 (0.88 to 1.39)
Central nervous system problems	0	3.9	1.23 (1.13 to 1.35)*
Prostatic disease	0	8.4	1.24 (1.13 to 1.36)*
Hormonal (hypogonadism, hyperprolactinaemia)	0	2.8	1.23 (1.11 to 1.36)
Number of comorbidities per patient			
At least one comorbidity	52.2	61.5	1.07 (0.90 to 1.28)
At least two comorbidities	13.0	36.5	1.21 (1.05 to 1.40)*
At least three comorbidities	4.4	14.4	1.17 (1.00 to 1.34)*
At least four comorbidities	0	5.8	1.23 (1.13 to 1.35)*
At least five comorbidities	0	1	1.22 (1.13 to 1.33)*

Data presented as median (interquartile range) or percentage. The star (*) denotes statistical significance.

Traditional cardiovascular risk factors such as arterial hypertension, diabetes mellitus, coronary heart disease, hypercholesterolaemia and smoking were not more prevalent in men with ED. Significantly more men with ED (13.8%) than those without ED (0%) consumed alcohol in excess of 2 units per day and twice as many had depression (not significant). Men with severe ED (IIEF-5 scores 5 to 7) had a low prevalence of alcoholism (5.7%), but the highest prevalence of depression as judged by the treating physician (10.8%). Central nervous system dysfunction was reported only in men with ED, in which it consisted of stroke, multiple sclerosis and dementia. More men with ED than those without ED had prostatic disease, whereas endocrine or medication-related factors did not differ between both groups.

### Demographics, disease characteristics and predictors of ED

**Figure 2:**
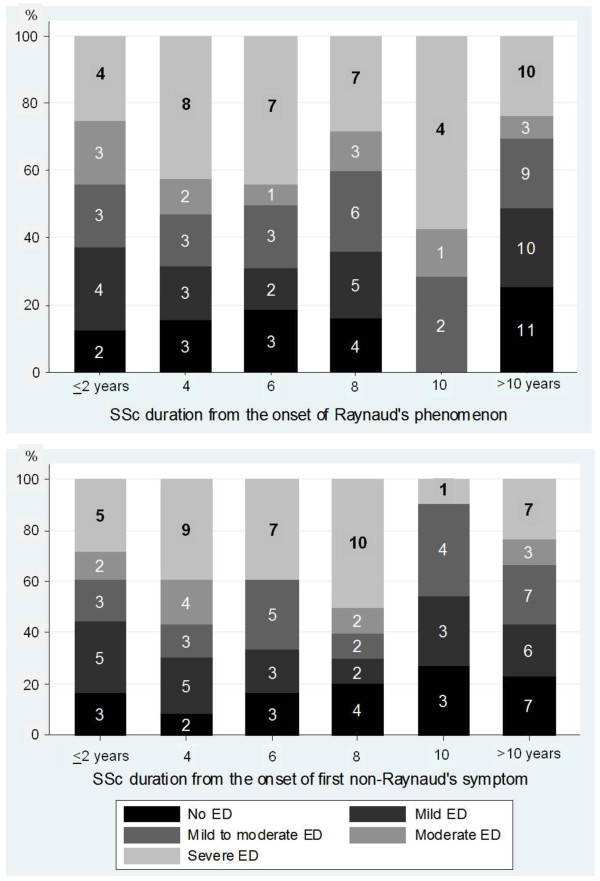
**Severity of erectile dysfunction as a function of disease duration**. Figures in bars represent the number of men within each subgroup; *y *axis, cumulative percentages. ED, erectile dysfunction; SSc, systemic sclerosis.

Patients with ED were significantly older than subjects without ED (Table 3). The median SSc duration was similar in both groups (approximately 7 years if measured from the onset of Raynaud's phenomenon, and 6 years if determined from the first non-Raynaud's symptom). The median duration of ED was 1.8 years (IQR 0.3 to 4.9). Patients with more severe ED had experienced erectile problems for a longer time (median of 4 years in patients with IIEF-5 score ≤ 7) than those with less severe ED. In the majority of patients, the erectile problem started after the onset of SSc (in 90.1% of SSc patients after the onset of Raynaud's phenomenon, and in 82.1% of men after the manifestation of the first non-Raynaud's symptom of SSc). The median time interval from the onset of the first non-Raynaud's symptom of SSc to the onset of ED was 4.1 years (IQR 1.5 to 8.3 years). An analysis by ED duration revealed a negative correlation between IIEF-5 score and time of ED *(P = *0.03). The IIEF-5 score was not correlated with SSc duration, as measured either from the onset of Raynaud's phenomenon or from the onset of first non-Raynaud's symptom, however, and about one-fifth of all men have maintained normal erections many years after SSc onset (Figure 2).

**Table 3:** Comparison of participants with and without ED

	No erectile dysfunction (*n *= 23)	Erectile dysfunction (*n *= 105)	Odds ratio (95% confidence interval)
IIEF-5 score	23 (22 to 25)	11 (5 to 16)	*P *< 0.001*
Age (years)^a^	45 (35.1 to 51.8)	55.7 (47.1 to 62.9)	*P *< 0.001*
SSc duration by Raynaud's phenomenon (years)	7.2 (4.3 to 15.3)	7.0 (3.4 to 11.6)	*P *= 0.44
SSc duration by first non-Raynaud's symptom (years)	6.6 (4.3 to 11.8)	5.6 (2.8 to 9.8)	*P *= 0.41
Topoisomerase I (Scl70)-positive	47.6	45.1	0.98 (0.80 to 1.19)
ACA-positive	5.0	18.8	1.21 (1.02 to 1.44)*
U1RNP-positive	0	5.9	1.25 (1.12 to 1.39)*
RNA polymerase III-positive	0	3.5	1.20 (1.08 to 1.33)*
Diffuse SSc	50	65.9	1.13 (0.92 to 1.40)
Puffy hands	57.1	52.9	0.97 (0.80 to 1.16)
mRSS	7 (2 to 19)	14 (8 to 23)	*P *= 0.05*
mRSS > 20	15.8	26.8	1.13 (0.91 to 1.40)
C-reactive protein elevation	14.3	25.3	1.13 (0.93 to 1.37)
Raynaud's phenomenon	90.5	95.5	1.22 (0.69 to 2.17)
Digital ulcers	47.6	41.9	0.95 (0.79 to 1.16)
Synovitis	19.1	19.5	1.01 (0.80 to 1.27)
Joint contractures	19.1	33.3	1.14 (0.95 to 1.36)
Tendon friction rubs	4.8	10.3	1.13 (0.90 to 1.42)
Muscle weakness	9.5	20.7	1.15 (0.96 to 1.38)
Muscle atrophy	4.8	18.4	1.21 (1.03 to 1.42)*
Creatine kinase elevation	19.1	12.2	0.88 (0.62 to 1.25)
Oesophageal symptoms	57.1	59.1	1.02 (0.84 to 1.23)
Stomach symptoms	19.1	12.5	0.90 (0.65 to 1.23)
Intestinal symptoms	4.8	15.9	1.19 (0.99 to 1.41)
Lung fibrosis on chest X-ray or HRCT	42.9	56.9	1.13 (0.87 to 1.47)
Forced vital capacity (% predicted)	95 (87 to 107)	91 (78 to 192)	*P *= 0.52
Forced vital capacity < 80%	9.5	25.7	1.26 (1.01 to 1.56)*
DLCO (% predicted)	81 (73 to 91)	66 (53 to 82)	*P *= 0.08
DLCO < 80%	42.9	63.6	1.24 (0.95 to 1.61)
PAPsys (mmHg)	23.5 (0 to 28)	29 (22 to 37)	*P *= 0.04*
PAPsys > 40 mmHg	0	24.4	1.32 (1.17 to 1.50)*
Diastolic dysfunction	15.8	17.3	1.02 (0.79 to 1.33)
Pericardial effusion	5.6	4.4	0.95 (0.53 to 1.68)
Left ventricular ejection fraction < 60%	27.8	18.3	0.88 (0.65 to 1.20)
Renal crisis	0	4.6	1.25 (1.14 to 1.38)*
Proteinuria	19.1	6.0	0.68 (0.37 to 1.22)
EULAR SSc activity score	1.0 (0.5 to 2.0)	2.5 (1.0 to 3.5)	*P *= 0.02*
High SSc activity (score ≥ 3)	11.8	43.4	1.38 (1.09 to 1.75)*
Hypocomplementaemia	5.0	6.3	1.05 (0.72 to 1.52)

Data presented as median (interquartile range) or percentage unless indicated otherwise. DLCO, diffusion capacity of the lung for carbon monoxide; HRCT; IIEF-5, International Index of Erectile Function-5; mRSS; modified Rodnan skin score; PAPsys, systolic pulmonary arterial pressure; SSc, systemic sclerosis. ^a^Age presented as mean + standard deviation (minimum to maximum). The star (*) denotes statistical significance

A total 52.4% of men without ED had one of the antinuclear antibodies typically tested for SSc; for example, antibodies directed against topoisomerase I (Scl70), centromere, U1 RNP and RNA polymerase III. Among men with ED these typical antinuclear antibodies were more prevalent (69.2%) than in men without ED (52.4%), but the difference was not statistically significant. The prevalence of autoantibodies against topoisomerase I (Scl70) was similar in the ED group and the non-ED group, but antibodies directed against centromere, U1RNP and RNA polymerase III were more frequent with ED (Table 3).

The presence of ED was also associated with more severe organ involvement in SSc. Men with any form of ED had a higher modified Rodnan skin score, and more frequently had muscle atrophy, a history of renal crisis, elevated pulmonary arterial pressure and restrictive lung disease (Table 3). Men with ED also had higher EULAR SSc activity scores than men with normal erectile function [13]. On multivariate analysis, however, only age remained a predictor of ED (*P *= 0.02, *R*^2 ^= 0.42).

We also performed an analysis of organ involvement by ED severity (Table 4). In this analysis, older age (*P *< 0.001) and impaired pulmonary function (*P *= 0.006 for normal forced vital capacity, *P *= 0.01 for forced vital capacity < 80% of normal) were associated with ED severity - indicators of pulmonary hypertension and SSc activity.

**Table 4:** Organ involvement by severity of erectile dysfunction

	Erectile dysfunction severity				
		
	Mild (*n *= 25)	Mild to moderate (*n *= 26)	Moderate (*n *= 14)	Severe (*n *= 40)	*P *value
IIEF-5 score	19 (18 to 20)	14 (13 to 16)	10 (8 to 11)	5 (5 to 6)	< 0.001*
Age (years)	50.5 (45.3 to 61.0)	54.1 (47.1 to 63.9)	54.9 (48.6 to 62.0)	57.3 (48.8 to 64.0)	< 0.001*
Duration of erectile dysfunction (years)	1.0 (0.6 to 2.3)	1.2 (0.1 to 2.6)	2.8 (0.1 to 5.0)	4.0 (1.0 to 6.4)	0.08
Diffuse SSc	59.1	47.6	72.7	82.1	0.08
C-reactive protein elevation	9.1	18.2	41.7	37.4	0.06
Forced vital capacity (% predicted)	99 (86 to 108)	100 (91 to 108)	84 (78 to 90)	81 (66 to 98)	0.006*
Forced vital capacity < 80%	16.7	6.3	33.3	47.6	0.01*
DLCO (% predicted)	70 (53 to 81)	79 (61 to 90)	64 (49 to 69)	56 (45 to 74.5)	0.02*
PAPsys (mmHg)	32 (25 to 38)	25 (21 to 29)	27 (19 to 36)	35 (25 to 39)	0.006*
PAPsys > 40 mmHg	25	10.0	16.7	38.5	0.02*
EULAR SSc activity score	1.5 (0.5 to 3.0)	2.5 (1.0 to 4.5)	3.5 (3.0 to 5.5)	2 (0.5 to 3.5)	0.047*
High SSc activity (score > 3)	26.7	47.1	83.3	40.0	0.02*

Data presented as median (interquartile range) or percentage. DLCO, diffusion capacity of the lung for carbon monoxide; IIEF-5, International Index of Erectile Function-5; PAPsys, systolic pulmonary arterial pressure; SSc, systemic sclerosis. *Significant at *P *< 0.05.

### Treatment of erectile dysfunction

Treatment information was obtained in 101 of the 105 men with ED (Table 5). A total 72.2% of men with abnormal erections did not receive any treatment for ED. In the remaining 27.8% of men, ED was treated with a phosphodiesterase-5 inhibitor as the recommended first-line modality in the non-SSc population. Sildenafil was the agent most commonly used; seven of the 15 men using sildenafil also had concomitant pulmonary arterial hypertension. Tadalafil was used in a total of 11 men. The proportion of patients with tadalafil and concomitant pulmonary arterial hypertension was not captured because the study was launched prior to the approval of tadalafil for pulmonary arterial hypertension. Two men with moderate ED were treated with intracavernous alprostadil injections. Three of 101 men with ED (IIEF-5 scores of 10, 16 and 20) received combination therapy. One man was treated with sildenafil plus vardenafil, one man received sildenafil plus tadalafil and one man received sildenafil plus intracavernous alprostadil.

**Table 5:** Treatment of erectile dysfunction

	Erectile dysfunction severity				
	
	Mild (*n *= 25)	Mild to moderate (*n *= 26)	Moderate (*n *= 14)	Severe (*n *= 40)	All (*n *= 105)
Sildenafil	20	8	31	11	15
Tadalafil	12	8	15	11	11
Vardenafil	0	4	0	0	1
Alprostadil urethral	0	0	0	0	0
Alprostadil cavernous	0	0	15	0	2
Vacuum device	0	0	0	0	0
Penile prosthesis	0	0	0	5	2

Data represent percent of men on each treatment modality. A total of 3 men received combination therapy.

Other second-line treatments for ED, such as intraurethral alprostadil applications or vacuum devices, were not used. Two patients had received a penile prosthesis for severe ED; one patient had a normal IIEF-5 score after this procedure. The ED in the second patient who also suffered from multiple sclerosis had not improved from the otherwise uneventful prosthesis implantation.

## Discussion

Connective tissue diseases more frequently affect women and most studies have not addressed medical problems specific to men. This study represents the largest investigation so far of ED in men with SSc. The prevalence of ED in our survey is similar to, or even exceeds, the estimates from smaller studies [3,4] and is considerably higher than in the general population. A study in Massachusetts, for example, calculated the prevalence of complete impotence as 5 to 15% in men between 40 and 70 years of age in the general population [14]. The prevalence of ED in our study also exceeds estimates in other chronic disease populations, such as in diabetes mellitus (37 to 75%) [15,16], stroke (48%) [17], and arterial hypertension (23 to 46%) [18-20]. For rheumatoid arthritis the reported prevalence was 48% [4].

Patients with SSc not only have an elevated prevalence of ED, they also have more severe ED compared with the general population. The average IIEF-5 score in our study was 13.3, which is similar to the only other study in which ED severity was investigated in 17 men with SSc [6]. In comparison, the average IIEF-5 score in a non-SSc population with a similar age was 21.3 [11]. About one-third of men with SSc had severe ED in our investigation, whereas in the general population only 8.5% of the men with ED reported moderate or severe ED [11]. In men with non-SSc causes of ED - for example, diabetes [15,16], arterial hypertension [18-20], and stroke [17] - the severity of ED was also milder than in SSc.

Although ED manifests after SSc onset in the vast majority of men [4], it appears as a relatively early symptom of SSc with a mean delay from SSc diagnosis of 2.7 years [4]. ED will probably not become a diagnostic predictor of SSc, given the fact that ED mostly follows SSc onset. This contrasts with the role of ED in the general population, in which ED is an important harbinger of subsequent cardiovascular disease [8,21].

Our study confirms age as an important but nonmodifiable risk factor for ED development in SSc [6]. More importantly, our findings show an association with SSc severity in terms of restrictive lung disease and renal and pulmonary vasculopathy. Our study also examined for the first time the relationship between ED and autoantibody status, but failed to identify a protective antibody or an antinuclear antibody conferring an elevated risk of ED development. Among the modifiable risk factors of ED, the elevated alcohol consumption of men with ED deserves attention. The present data, however, do not permit one to differentiate whether alcohol consumption is a cause of ED, is a coping strategy for ED, or is unrelated to ED. Although the ED was more frequent in SSc men than in the normal population and age was an important risk factor for ED, the interpretation of the SSc effect in our study would be facilitated by the recruitment of a non-SSc control group matched for known ED risk factors.

Treatment guidelines for ED in the general population suggest that modifiable risk factors such as lifestyle, psychological or drug-related factors be minimised prior to or in conjunction with specific ED therapy [12,22]. In our study, about one-fifth of SSc patients had at least one such modifiable comorbidity. A higher proportion of men with SSc-related ED than those without ED had more than two comorbidities, indicating that these factors may contribute to the development of ED not only in the general population but also in patients with SSc and that these factors should be aggressively addressed. In the non-SSc population, pharmacotherapy with phosphodiesterase-5 inhibitors is recommended as first-line specific treatment [22]. In SSc, the efficacy data of phosphodiesterase-5 inhibitors for ED with on-demand sildenafil were disappointing [23], whereas the longer-acting tadalafil is slightly better evaluated [24,25]. Second-line and third-line treatment options such as vacuum devices or intracavernous or intraurethral applications of alprostadil were used by only a minority of men with SSc, and a similar minority was equipped with a penile prosthesis although successful implantations were previously reported [7].

Our study has both strengths and limitations. It represents the largest analysis of impotence in men with SSc. The multicentric nature of our investigations may, on the one hand, be more representative of all men affected by the disease than a monocentric study, but on the other hand may lead to difficulties in standardising data collection. Although centres were asked to recruit men consecutively, there is always a risk of recruitment bias, as indicated by the slight differences observed between participants and non-participants. Depression was only judged by the treating physician and not captured with a validated questionnaire. Lastly, it would have been interesting to correlate the prevalence of ED with changes on nailfold capillaroscopy but these data were not available in the majority of patients.

## Conclusion

Our study indicates that ED is a common, severe and early problem in men with SSc. ED is associated with a higher age of patients and the presence of restrictive lung disease, as well as with renal and pulmonary vasculopathy. The reasons for the overall low treatment coverage were not the assessed in this study but clearly a heightened awareness among physicians and more research into pathophysiology, longitudinal development, treatment and psychosocial impact are urgently needed.

## Abbreviations

ED: erectile dysfunction; IIEF-5: International Index of Erectile Function-5; IQR: interquartile range; EUSTAR: EULAR Scleroderma Trial and Research; SSc: systemic sclerosis.

## Competing interests

The authors declare that they have no competing interests.

## Authors' contributions

UAW participated in the design of the study and statistical analysis and prepared the manuscript. CF performed the statistical analysis and helped to draft the manuscript. AT and TH participated in the design of the study and helped to draft the manuscript. All other coauthors participated in the data acquisition and helped to draft the manuscript. All authors read and approved the final manuscript.
